# Water against stone: *PLOS Global Public Health*’s persistence in the pursuit of equity in public health

**DOI:** 10.1371/journal.pgph.0006362

**Published:** 2026-05-19

**Authors:** Julia Robinson, Manuela De Allegri, Catherine Kyobutungi

**Affiliations:** 1 Public Library of Science (PLOS), San Francisco, California, United States of America; 2 MDA, Heidelberg Institute of Global Health, Medical Faculty and University Hospital, Heidelberg University, Heidelberg, Germany; 3 African Population and Health Research Center, Nairobi, Kenya; PLOS: Public Library of Science, UNITED KINGDOM OF GREAT BRITAIN AND NORTHERN IRELAND

To work in the field of public health is to work in a field of constant flux, with a constant need to adapt. Some of these changes can be predicted using the tools of clinical science and epidemiology – we know a great deal about infection vectors, for instance, or the impact of vaccination rates on probable transmission and can design population health programs to address these in advance. Some of these changes are less likely to be predicted, such as the health impact of a novel virus or a sudden natural or manmade disaster.

We launched *PLOS Global Public Health* in 2021 in a time of great societal, political, and cultural change; as we have written before [1], the journal was born in a time of rampant COVID transmission and spread, when movements such as Black Lives Matter and #MeToo and efforts to decolonize global health were at the forefront of our minds and our work. The fight for vaccine equity, for instance – in 2021, people in high-income countries were receiving their second or third booster for COVID, whereas most countries in Africa were unable to access even an initial dose [2]. This was a clear continuation of a legacy of neglect that many of us working in global health were so incessantly working to overturn.

We could not have known when we launched the journal five years ago that we’d find ourselves in the current world, where the field of global health and development as we had known it would have been once again upended by the current US administration. As it has been well-documented, in the pages of *PLOS Global Public Health* [3] and elsewhere [4], the events of 2025 saw the sudden, cruel and drastic elimination of funding and support of health programs around the world, as well as threats to human rights, the public trust of science and medicine. These have sent shock waves across the sector. At the same time, many are wondering if this inflection point is a moment for global public health to truly free itself from the hegemony of rich countries dictating other countries’ priorities - can global health rise from the ashes truly transformed? Will this be the moment where low- and middle-income (LMIC) nations take on the leadership role and chart their own destiny [5]? As we witness the current chaos of world politics, healthcare workers, program implementers, academics, students, activists, many of us have grappled with the question of, what do we do now?

Health challenges - whether rising rates of chronic conditions, mental health pressures, infectious outbreaks, or the impacts of climate change - tend to echo across borders, reminding us that no country is immune to the vulnerabilities of modern health systems. Indeed, the COVID pandemic and its aftermath laid this bare in incredibly stark terms, magnifying disparities in access to health care as well as in health outcomes [6]. Underinvestment, fragmentation, and governance constraints continue to characterize most health systems, limiting capacity to absorb and adapt to a changing environment.

Yet the ways nations respond to these shared problems can differ dramatically, shaped by political priorities, resources, culture, and community structures. These differences are not just interesting; they’re instructive. When countries learn from one another’s successes and missteps, they generate insights that can inform smarter policies and better outcomes elsewhere. In a world where health threats are increasingly interconnected, the exchange of ideas and evidence across countries becomes not just beneficial, but essential for building stronger, more resilient health systems. Cross-country exchange and learning are instrumental in fostering evidence-based policymaking and through active promotion of international collaborations, can serve to empower local and global leadership.

The events of the past year have raised new questions about the place of this journal in the global knowledge system. As the world reeled from significant disruptions in large parts of what has been a feature of the global health architecture – the dismantling of USAID and the attendant international non-governmental organization (INGO) ecosystem, the withdrawal from and of funding for several multilateral agencies active in global health and the resulting enormous disruptions in the lives, livelihoods, and health outcomes of real communities - new policy shifts among the biggest funders for global health have created new challenges and opportunities.

On the one hand, the current crisis is being viewed as an opportunity to assert greater agency over health priorities and to reshape health systems on more sovereign and locally defined terms. On the other hand, as global health architecture is reshaped and resources are constrained, public health is being repositioned not as a shared global commitment, but as an investment portfolio requiring performance justification. The language of return on investment (ROI) – especially holding the funder’s perspective - has increasingly displaced earlier commitments to equity and solidarity in framing the value of public health.

When ROI becomes the dominant lens, public health risks drifting toward what is profitable rather than what is equitable or necessary. This can disadvantage and further entrench the inequalities faced by marginalized groups, whose needs may not generate high economic returns but are central to justice and population well‑being. In short, ROI can be a useful tool, but if it becomes the compass – which it seems to be becoming in this modern age of public health austerity - it can pull public health away from its core mission of protecting and promoting health for everyone.

Moreover, in a world which has witnessed substantial cuts to global health funding, the risk is not trivial for those of us working in public health to be asked to assess the return adopting the perspective of those who have made the investment, frequently research agencies and/or development partners located in high-income countries. Such an approach risks reversing all the gains and the continuous efforts made over the years towards decolonizing global health and the gains we have made in integrating social justice, human dignity, and diversity, equity and inclusion in our field.

The three of us are seeking answers, just like everyone else. As public health researchers and activists, our hearts break for the turmoil and uncertainty around us, for the people whose treatments have abruptly been stopped, for the health workers and public health staff and community workers who suddenly find themselves without work, for students graduating into a chaotic world where leaders are retreating into nationalism, populism and authoritarianism. But what we can do is to go back to the place we started and recognize the importance of spaces where allies can join together and uphold the foundational values of a more just world. These values include respect for all humans and their rights, compassion for everyone, and equity. We recognize that “global” health is (and always has been) practiced locally. We will continue to promote equity-oriented research and to encourage submissions addressing public health concerns of historically marginalized groups across the globe – whether these health disparities are happening in high-income countries, low-income countries, or anywhere in between.

As a journal we may not have the political traction to change the course of history. Political commitment is an essential element needed to translate a new vision of global public health into practice. Yet we should not underestimate the power of written words in preserving ideas over time, shaping beliefs and attitudes, and ultimately also influencing action toward more equitable and inclusive health policies. We can be like a water drop against a stone: patient, persistent, and unstoppable. We trust that by working as a community to promote equity-oriented and inclusive global public health research, we can reposition social justice as the core value guiding our commitment to global public health. In the years that come, true to the commitments we made in our launch editorial [7], we wish to do more than just re-imagine global public health. We want to translate this vision into practice.
